# Führung von und in Familienunternehmen: Eine theoriebezogene Standortbestimmung

**DOI:** 10.1007/s11612-021-00609-2

**Published:** 2021-11-02

**Authors:** Rudolf Wimmer

**Affiliations:** 1 osb Wien Consulting GmbH, Volksgartenstraße 3/1. DG, 1010 Wien, Österreich; 2 Private Universität Witten/Herdecke, Witten, Deutschland

**Keywords:** Familienunternehmen, Führungstheorien, Principal Agent Ansatz, Stewardship-Theorie, Social-emotional-wealth (SEW)-Ansatz, Systemtheoretisches Verständnis von Führung, Family companies, Leadership theories, Principal agent approach, Stewardship theory, Social-emotional wealth (SEW) approach, Systemic approach to leadership (systems theory)

## Abstract

Dieser Beitrag der Zeitschrift „Gruppe. Interaktion. Organisation. (GIO)“ geht davon aus, dass die etablierte Führungsforschung die spezifische Führungspraxis in familiengeführten Unternehmen bislang weitestgehend ignoriert. Das ist erstaunlich, sind doch weltweit, je nach Region unterschiedlich, 70–90 % der Unternehmen diesem Typus zuzurechnen. Auf der anderen Seite hat zur Zeit die stark expandierende Forschung zu den Besonderheiten von Familienunternehmen das Thema Führung auch nur sehr indirekt am Schirm nämlich als Einflussnahme der Eignerfamilie auf die Unternehmensentwicklung. Die in dieser Forschung dominierenden Theoriezugänge werden in der vorliegenden Arbeit mit Blick auf ihre Erklärungskraft bzw. auf ihre paradigmatischen Begrenzungen rekonstruiert und dem Differenzierungsvermögen eines systemtheoretischen Verständnisses von Führung und Organisation gegenübergestellt.

Ziel dieser Gegenüberstellung ist es, den theoretischen Blick auf die Besonderheiten des Führungsgeschehens in Familienunternehmen zu schärfen.

## Abstract (en)

Up to now, the established leadership research has widely ignored the specific leadership practices in family-run companies. This is quite astonishing given the fact that today 70–90% of all companies worldwide (regional variations considered) belong to this type of enterprises. On the other hand, the currently strongly expanding research on the peculiarities of family companies touches the topic of leadership only in a very implicit way when dealing with the influence of the owner family on the future corporate development. In this article, we present the leading theoretical approaches in this special field of research, in particular with a view to their explanatory power as well as their paradigmatic limitations in comparison to the possibilities of a systemic approach to leadership and organization.

The aim of this comparison is to sharpen the theoretical perception of the peculiarities of Leadership & management in family-run enterprises.

## Familiengeführte Unternehmen – ein blinder Fleck in der Führungsforschung

Das Thema Führung (heute spricht man lieber von Leadership) besitzt aktuell gerade wieder Hochkonjunktur. Es schiebt sich in der gesellschaftlichen Aufmerksamkeit in periodischen Abständen immer wieder mal ganz nach vorne. Aktuell sind es die weit verzweigten Begleiterscheinungen der digitalen Transformation, die nicht mehr zu leugnenden ökologischen Gefährdungslagen unserer Gesellschaft und obendrein die Covid-19 Pandemie, die die verantwortlichen Entscheidungsträger in Politik, Wirtschaft und Gesellschaft vor bislang nicht gekannte Herausforderungen stellen. Da wird der Ruf nach „guter“ Führung, nach neuen wirksamen Lösungen, die die Unsicherheit wieder wegnehmen, unüberhörbar.

Im Unternehmenskontext sind dies immer Zeiten, in denen neue Konzepte und Lösungsversprechen ins Leben treten und vorübergehend viel Aufmerksamkeit auf sich ziehen. Im Moment ist es das Zurückschrauben und Abflachen von Hierarchie in Verbindung mit agileren, teamförmigen Arbeitsformen unterstützt durch „collaborativ“ Leadership, die in den einschlägigen Publikationen und Diskussionsforen gerade die Hoheit über die managerialen Stammtische zu gewinnen versuchen (beispielhaft Glatzel und Lieckweg 2020). Diese „innovativen“ Lösungsangebote bilden vielfach den Stoff für das Entstehen von neuen Managementmoden, die für eine gewisse Zeit die Veränderungsanstrengungen in Unternehmen anleiten (dazu nach wie vor lesenswert Kieser 1996).

Lassen sich familiengeführte Unternehmen von solchen Entwicklungen beeindrucken? In der Regel nicht. Familienunternehmen repräsentieren in der Art, wie sie geführt werden eine ganz eigene Welt. Diese Welt kommt in der weit verzweigten und ganze Bibliotheken füllenden Führungsforschung der letzten Jahrzehnte allerdings nicht ernsthaft vor. Für diesen blinden Fleck der Forschung gibt es gute Gründe.

Diese sind wohl in den spezifischen Besonderheiten dieses Unternehmenstyps zu sehen. Dass sich familiengeführte Unternehmen in wesentlichen Dimensionen von Nichtfamilienunternehmen unterscheiden, in dieser Einschätzung ist sich die einschlägige Forschung inzwischen einig (vgl. etwa Hack 2009; sowie Wimmer 2021). Das Anderssein dieses Unternehmenstyps resultiert im Wesentlichen aus dem Umstand, dass auf der Seite des Eigentums eine Familie bzw. eine Mehrfamilienkonstellation die unternehmerische Verantwortung trägt. Dieses Hineinwachsen einer Familie in diese spezifische Verantwortung, die solche Familien erst zu einer Unternehmerfamilie macht, passiert in der Regel in der Pioniergeneration, in der die Weichen für eine transgenerationale Langzeitperspektive der Unternehmensentwicklung und der sie tragenden Eigentumsverhältnisse gestellt werden. Mit dieser Weichenstellung startet jene charakteristische Koevolution von Unternehmen und Eignerfamilie, die auf eine höchst individuelle Weise jedes dieser Unternehmen mit einer gewissen Familienhaftigkeit ausstattet und zur gleichen Zeit in der Familie Eigentümlichkeiten entstehen lässt, die es rechtfertigen, hier von einem eigenständigen Familientypus zu sprechen (vgl. dazu Wimmer und Simon 2019). Eine der identitätsstiftenden, familial geprägten Besonderheiten solcher Unternehmen ist zweifelsohne die Art und Weise, wie in ihnen Führung organisiert ist und alltäglich praktiziert wird.

Die vorliegende Arbeit geht zunächst in aller Kürze diesen Besonderheiten vor allem in jener Governancesituation nach, in der Führung und Eigentum noch in einer Hand vereint sind. Diese eignergeprägte Führungskonstellation ist nach wie vor für den allergrößten Teil der Familienunternehmen kennzeichnend (vgl. Stiftung Familienunternehmen 2019). Vor diesem Hintergrund werden dann jene führenden Theoriezugänge rekonstruiert, die heute in der Familienunternehmensforschung überwiegend genutzt werden, um das führungsbezogene Entscheidungsgeschehen in Familienunternehmen beschreibbar zu machen. Angesichts der paradigmatischen Verengungen dieser Zugänge wird abschließend der Versuch unternommen, diesen ein systemtheoretisches Verständnis gegenüberzustellen, um zu überprüfen, ob damit ein zusätzlicher Erkenntnisgewinn erzielt und damit eine Anreicherung der Theoriebildung bezogen auf diesen Unternehmenstyp geleistet werden kann.

## Patriarchale Führungsmuster und ihre Konsequenzen

Der alles entscheidende Unterschied zur Denkwelt über Führung und Organisation in Nichtfamilienunternehmen liegt wohl in dem Umstand, dass an der Spitze von Familienunternehmen in der Regel Unternehmer und Unternehmerinnen stehen, die ihrem unternehmerischen Engagement einen langfristigen Zeithorizont zugrunde legen, um so das Unternehmen erfolgreich, d. h. wirtschaftlich gestärkt in die nächste Generation zu bringen. Strukturell kommt das Unternehmerische hier besonders dadurch zum Ausdruck, dass an der Unternehmensspitze drei ganz unterschiedliche Funktionen in einer Hand vereint sind: die des Eigentums am Unternehmen, die der Führung desselben und die des Oberhaupts der Unternehmerfamilie. Diese Einheit an der Spitze des Unternehmens, die regelmäßig noch durch eine persönliche Haftung für die eingegangenen Risiken unterstrichen wird, lässt üblicherweise in den frühen Phasen im Lebenszyklus dieser Unternehmen ganz eigentümliche, unverwechselbare Führungs- und Organisationsverhältnisse wachsen, eingebettet in eine dazu passende, stark wertegetriebene Organisationskultur.

Das hervorstechendste Merkmal dieser Funktionseinheit besteht darin, dass sie der Unternehmensspitze eine außergewöhnliche Autoritätsposition verschafft. Wenn der unternehmerische Start erfolgreich gelungen ist, dann bündelt und festigt sich an der Spitze bei allen Beteiligten die Erwartungssicherheit, dass dort eine gesamthafte Verantwortungsübernahme für alle auftauchenden Unsicherheiten und die damit verbundenen Entscheidungsnotwendigkeiten wirksam unterstellt werden kann. Mit dieser weitreichenden Verantwortungsbündelung geht in der Regel eine klare Arbeitsteilung Hand in Hand. Alles, was irgendwie mit Führungsaufgaben, d. h. mit im Alltag anfallenden Entscheidungserfordernissen verknüpft ist, wird von der Spitze erwartet und ist dort zumeist auch gut versorgt. Der Rest der Organisation kann sich auf die Umsetzung dieser Entscheidungen und die Erledigung all dessen, was arbeitsmäßig gerade ansteht, konzentrieren. In diesem Zusammenspiel erübrigt es sich, feinziselierte hierarchische Ebenen auszudifferenzieren und die Führungsverantwortung sorgfältig abgestuft auf viele Schultern zu verteilen. Deshalb sind in solchermaßen geführten Unternehmen keine formell festgelegten und allgemein verbindlich kommunizierten Organigramme anzutreffen. Flache Hierarchien bilden hier üblicherweise den Normalzustand. Letztlich gibt es nur eine wirklich wichtige Führungsbeziehung und das ist die zum Inhaber, zur Inhaberin an der Unternehmensspitze, auch wenn es da in der Praxis je nach Unternehmensgröße natürlich große Abstufungen gibt. Es ist dies eine Relation, in die fast immer auch eine persönlich getönte, emotionale Seite miteingebaut ist. Die Verantwortlichen an der Spitze versuchen deshalb, solange es irgendwie geht, durch das Herstellen und Aufrechterhalten von persönlichen Kontakten zu den Beschäftigten, den mit einer solchen Führungskonstellation verbundenen emotionalen Erwartungen tatsächlich auch gerecht zu werden.

Hinsichtlich des Organisationsaufbaus bedeuten solche Führungsverhältnisse, dass im Zuge des Wachstums, wenn eine klarere organisatorische Aufgabenteilung unvermeidlich wird, diese Aufgabenzuordnung um langgediente, bewährte und vertrauenswürdige Leistungsträger herum erfolgt. So entstehen im Laufe der Zeit organisationsintern Subeinheiten und Bereiche, deren genauen Aufgabenzuschnitt man nur verstehen kann, wenn man sieht, um welche Personen herum aus welchem Anlass dieser Zuschnitt geformt worden ist. In diesem Organisationsmuster folgen die Aufgaben und die damit verbundenen Verantwortlichkeiten den Personen und nicht umgekehrt. So entsteht im Zuge der Wachstumsentwicklung des Unternehmens um die Spitze herum ein größer werdender Kreis an langgedienten Verantwortungsträgern, die sich auf ein besonderes Vertrauens- und Naheverhältnis zum Inhaber stützen können und in ihrer Summe die wesentlichen Funktionsbereiche repräsentieren. Die alltägliche Zusammenarbeit funktioniert ohne aufwendige Abstimmungsprozesse und ohne formal festgelegte Strukturen der Regelkommunikation. Der Bedarf an bürokratischen Regelwerken zur Steuerung des operativen Arbeitsgeschehens wird, soweit es irgend geht, möglichst geringgehalten. Diese Form des regelarmen, kommunikationssparenden Miteinanders ist möglich, weil man sich wechselseitig dank der langen Zugehörigkeit sehr gut kennt und um die jeweiligen Stärken und Schwächen sehr genau Bescheid weiß und weil jeder darauf vertrauen kann, dass alle anderen auch ihre gesamte Energie in den Dienst des Unternehmens stellen und deshalb engagierte Kooperation im Sinne der gemeinsamen Sache als selbstverständlich erwartet werden kann.

Die Funktionstüchtigkeit solcher Führungsverhältnisse steht und fällt mit dem Vorhandensein einer starken Unternehmenskultur, die im Kern eine Handvoll von gemeinsam geteilten Grundüberzeugungen und Werthaltungen beinhaltet, die im Alltag das Verhalten untereinander koordinieren, ohne dass darüber viel geredet werden muss. Dazu zählt auf der einen Seite die tiefe Loyalität der Beschäftigten dem nachhaltigen Wohlergehen des Unternehmens und seiner Eignerfamilie gegenüber, eine Loyalität die regelmäßig zu einer außergewöhnlichen Einsatzbereitschaft führt. Dem steht auf der anderen Seite die Gewissheit der Leute gegenüber, dass sie bei den einschlägigen Unternehmensentscheidungen auch als Personen mit ihren je individuellen Besonderheiten und Problemlagen zählen. Wir haben es hier mit kulturell fest verankerten Werthaltungen im Geben und Nehmen zu tun, die in der Tendenz eher familialen Mustern entsprechen als einer kühlen ökonomischen Tauschlogik. Diese Prinzipien fundieren nichtökonomische Prämissen für die wechselseitigen Ausgleichserwartungen, in die das eigentliche Wirtschaften des Unternehmens eingebettet ist (vgl. dazu Wimmer 2021, S. 243 ff.).

Diese ganz spezifische familienhafte Reziprozität, die die kulturelle Stärke gut geführter Familienunternehmen ausmacht, ist das Fundament, auf dem sich die charakteristischen Führungspraktiken von Inhabern an der Spitze ihrer Unternehmen entfalten können. Weil diese Praktiken vieles von dem widerspiegeln, wie auch in Familien das Miteinander der Partner wie auch im Verhältnis zum Nachwuchs gesteuert wird, werden diese Muster von außenstehenden Beobachtern gerne als „patriarchalisch“ bezeichnet. Diese Begrifflichkeit bringt in unserem Zusammenhang keineswegs einen negativen bzw. kritischen Beigeschmack zum Ausdruck. Denn es ist gerade diese Familienhaftigkeit und die damit verbundenen Gestaltungsprinzipien des alltäglichen Miteinanders, die die vielfach bewunderte Leistungsfähigkeit dieses Unternehmenstyps ausmacht. (Für dieses Phänomen hat sich in der Forschung der Begriff „familiness“ eingebürgert, vgl. Habbershon et al. 2003; sowie Frank et al. 2010).

Die angesprochenen Leistungsvorteile, die mit der Einheit von Eigentum und Führung, also mit dem Unternehmertum an der Spitze von Unternehmen zumeist verbunden sind, sind in der Literatur vielfach beschrieben worden (vgl. Wimmer et al. 2018): Kurze Entscheidungswege, eine unternehmerisch kalkulierte Risikobereitschaft, ein sparsamer Umgang mit Ressourcen, verbunden mit einer langfristig ausgerichteten Investitionspolitik, eine hohe Umsetzungsgeschwindigkeit bei erkannten Handlungsnotwendigkeiten, eine konsequente Ausrichtung am Kundennutzen und vielfach dadurch angeregt eine kontinuierliche Innovation des eigenen Leistungsportfolios, ein fairer Umgang mit den erfolgskritischen Partnern in der eigenen Wertschöpfungskette, eine außergewöhnliche Einsatzbereitschaft der eigenen Belegschaft und dergleichen mehr, all dies zusammen sorgt unter ganz bestimmten Rahmenbedingungen für jene typischen Wettbewerbsvorteile, die eine überdurchschnittliche Ertragskraft und damit eine nachhaltige Wachstumsentwicklung ermöglichen. Dieses besondere Leistungsvermögen wird in der Literatur gerne unter dem „resource-based-view“ zusammengefasst (vgl. dazu Habbershon und Williams (1999), Rau (2014) sowie Sirmon und Hitt (2003), die dafür auf das spezifische „social capital“ von Familienunternehmen rekurrieren).

Der Blick auf die charakteristischen Erfolgsmuster familiengeführter Unternehmen darf jedoch gegenüber den spezifischen Selbstgefährdungspotenzialen und Entwicklungsdilemmata nicht blind machen, die in diese Praktiken unweigerlich eingebaut sind. Das was in einer bestimmten Phase besonders erfolgreich gemacht hat, kann unter veränderten Rahmenbedingungen in eine Existenzgefährdung umschlagen (zu dieser Janusköpfigkeit vgl. ausführlich Wimmer et al. 2018). Die besonderen Erfolgschancen der hier zur Debatte stehenden Führungskonstellation, die nach wie vor bei mehr als zwei Drittel vor allem der kleineren und mittelgroßen Familienunternehmen anzutreffen ist, sind an klar benennbare Voraussetzungen geknüpft, die gerade bei wachstumsstarken Familienunternehmen vielfach nicht mehr gegeben sind. Die Funktionstüchtigkeit dieser für eignergeführte Unternehmen typischen Führungskonstellationen ist auf Marktgegebenheiten angewiesen, die ein organisches Wachstum erlauben und zwar mit einer Geschwindigkeit und Dynamik, die unternehmensintern mit den eingeschwungenen Bearbeitungsmustern gut verdaut werden können. Diese interne „Verdaubarkeit“ ermöglichende Synchronizität von unternehmensinterner und externer Entwicklung ist angesichts einer spürbaren Veränderungsdynamik hin zu deutlich komplexer werdenden Verhältnissen an gut angebbaren Punkten im Lebenszyklus von Familienunternehmen nicht mehr erwartbar. Jene Familienunternehmen, die den dadurch angestoßenen Transformationsprozess erfolgreich bewältigen, verabschieden sich von den eher impliziten Führungsmustern der patriarchalen Welt und verlassen das Primat der Personenorientierung in der Gestaltung der organisationalen Binnenverhältnisse. Diese Veränderungen münden in deutlich komplexeren Führungskonstellationen, die an der Spitze Topmanagementteams vorsehen, in denen häufig Mitglieder aus der Familie mit familienfremden Verantwortungsträgern zusammenwirken. Solche Governancelösungen, die zumeist auch die Einrichtung von Aufsichtsgremien beinhalten, haben in der Regel auch einen weitreichenden Wandel im Selbstverständnis der daran beteiligten Unternehmerfamilien zur Folge. Alberto Gimeno et al. (2010) hat diesen Wandel durch die Beschreibung unterschiedlicher mentaler Modelle dieser Familien auf den Begriff zu bringen versucht. Wir vermeiden in unserem Kontext diese Begrifflichkeit, weil diese den angedeuteten Funktionswandel auf der Eigentümerseite zu einseitig als vorwiegend mentales Problem der handelnden Personen adressiert.

Wir gehen im nächsten Abschnitt nun dazu über, die in der Forschung primär genutzten Theorieressourcen zu rekonstruieren, um deren Erklärungskraft für die charakteristischen Führungsherausforderungen dieses Unternehmenstyps auf den Prüfstand zu stellen.

## Dominante Theorieansätze in der Familienunternehmensforschung, die sich auf Fragen der Führung und Steuerung dieses Unternehmenstyps fokussieren

Die Forschung zu Familienunternehmen ist ein vergleichsweise noch sehr junges Feld. Es hat sich in den zurückliegenden drei Jahrzehnten auf internationaler Ebene als eigenständige Subdisziplin der Wirtschaftswissenschaften erfolgreich ausdifferenziert und in der Zwischenzeit in der einschlägigen scientific community erstaunlich viel Aufmerksamkeit auf sich gezogen (für einen guten Überblick vgl. Sharma et al. 2014). Angesichts des Umstandes, dass bei diesem Typus von Wirtschaftsorganisation so unterschiedliche und in ihrer Logik so gegensätzliche soziale Formationen in einer einander prägenden Koevolution zusammenwirken (eine Familie auf der einen Seite und ein Unternehmen auf der anderen Seite) ist es nicht verwunderlich, dass für seine Erforschung ausgesprochen heterogene Theoriezugänge genutzt werden, um den Besonderheiten dieses Typs angemessen auf die Spur zu kommen. Diese paradigmatische Vielfalt mag man als ein Problem sehen und dieses dem noch geringen Reifegrad der noch jungen Disziplin zurechnen. Man kann sie aber auch als Spiegel der spezifischen Komplexität dieser weltweit dominanten Ausprägung wirtschaftlicher Aktivitäten sehen, deren Erforschung eben eine eigene komplexitätsadäquate „transdisziplinäre“ Theoriebildung benötigt (vgl. dazu etwa Kormann und Wimmer 2018). Die nun folgenden Überlegungen möchten dazu mit Blick auf das Thema Führung einen kleinen Beitrag leisten. In einem ersten Schritt werden drei in der Forschung aktuell sehr etablierte Theoriezugänge mit dem Ziel rekonstruiert, herauszuarbeiten, was man mit diesen „Brillen“ jeweils zu sehen bekommt und welche spezifischen Erkenntnisbegrenzungen andererseits mit diesen Theoriefestlegungen verknüpft sind. In einem zweiten Schritt wird ein Theorieangebot formuliert, das von sich behauptet, die tradierten Begrenzungen weitgehend hinter sich zu lassen und die Eigentümlichkeiten des Führungsgeschehens in Familienunternehmen realitätsgerechter zu erfassen in der Lage ist.

### Die Principal-Agent-Theorie und ihre Grundannahmen für die Steuerung und Funktionsweise familiengeführter Unternehmen

Der Principal-Agent-Ansatz wurzelt im Kern in den theoretischen Grundprämissen der „neueren Institutionenökonomik“ (dazu Richter und Furubotn 2003). Die institutionenökonomischen Ansätze gehen von Akteuren aus (Konsumenten, Haushalte, Unternehmern, etc.), die in ihren wirtschaftlichen Aktivitäten unvermeidlicherweise mit unvollkommenen Informationen operieren und dabei ganz grundsätzlich eine begrenzte Rationalität aufweisen. In ihrem Zusammenwirken mit anderen, d. h. in ihren wirtschaftlichen Austauschbeziehungen versuchen diese Akteure (hier der klassischen Annahme des „homo oeconomicus“ folgend) immer und überall ihren ganz persönlichen Nutzen zu optimieren, ihre eigenen Ziele und Interessen durchzusetzen und dies auf eine Art und Weise, die nach Möglichkeit die eigenen Risiken minimiert. Angesichts des Umstandes, dass das Realisieren wirtschaftlicher Interessen immer auf ein Mitwirken anderer Akteure angewiesen ist, die ihrerseits ihren eigenen Nutzen optimieren, sind wirtschaftliche Prozesse erwartbar, die von einer ganz charakteristischen Wettbewerbsdynamik rund um die Lösung von Knappheitsproblemen gekennzeichnet sind. Genau diese Dynamiken sind auf den verschiedenen Ebenen der Gesellschaft letztlich der bevorzugte Gegenstand wirtschaftswissenschaftlicher Forschung.

Die Principal-Agent-Theorie konzentriert sich aus diesem weiten Feld wirtschaftlicher Prozesse auf einen ganz bestimmten Zusammenhang, der letztlich die Grundlage für eine darauf basierende Theorie des Unternehmens bildet (vgl. Jensen 2003). Dieser Zusammenhang fokussiert auf die Situation eines Principals, der eine bestimmte Aufgabe mit einer klaren Zielsetzung erledigt haben will und damit jemand anderen (den Agenten) gegen eine definierte Entlohnung mit der Bewältigung dieser Aufgabe beauftragt. Aus dieser vertraglich begründeten Konstellation entstehen für einen Prinzipal angesichts der unterstellten Prämissen (z. B. persönliche Nutzenoptimierung und schwer zu behebende Informationsasymmetrien) gut angebbare Problematiken (sogenannte „agency problems“), deren genauere Analyse und möglichst effiziente Bewältigung Gegenstand der Forschung aus Sicht dieses Theoriezugangs sind (dazu grundlegend Eisenhardt 1989). Das Paradebeispiel für eine solche Kooperationssituation bildet das Verhältnis des Eigentümers zum Topmanagement seines Unternehmens, sobald es zu einer Trennung der Funktionen Eigentum und Unternehmensführung gekommen ist (vgl. Jensen und Meckling 1976). „Separation of ownership and management is a key component of agency theory“ (Madison et al. 2017, S. 46). Die Grundprobleme, die das spezifische Verhältnis des Principals zu seinem Agenten prägen, können unternehmensinternen neben dem bestimmenden Verhältnis der Eigentümer zu ihrem Management auch auf alle anderen Führungsbeziehungen angewendet werden (Eisenhardt 1989). Unternehmen lassen sich vor diesem Hintergrund als subtiles, vertragsbasiertes Geflecht ineinander verwobener Principal-Agent-Beziehungen begreifen und interpretieren. Sie stellen in dieser Perspektive eine organisationsspezifische Arena zur Verfügung, auf der die Akteure in der Verfolgung ihrer Interessen zwar voneinander abhängig sind, es aber im Grunde darum geht, die unvermeidlichen Informationsasymmetrien, die begrenzte Rationalität bzw. das spezifische Risikoverhalten der jeweils anderen in diesem Spiel für die eigene Nutzenoptimierung gezielt auszuschlachten.

Die mit dieser Führungskonstellation einhergehenden Herausforderungen lassen sich im Wesentlichen auf zwei Problemfelder zuspitzen. Zum einen ist davon auszugehen, dass der Agent in der Erfüllung der ihm übertragenen Aufgaben seine eigenen Ziele verfolgt, also ein opportunistisches Verhalten an den Tag legt und der Principal diese Abweichungen mangels realitätsgerechter Einblicke in das jeweilige Arbeitsgeschehen im Detail gar nicht beurteilen kann. Die aus diesem Umstand resultierenden Verhaltensmöglichkeiten des Agenten werden gerne unter dem Begriff des „moral hazard“ zusammengefasst (Eisenhardt 1989). Zum anderen hat der Agent immer die Möglichkeit, den Principal hinsichtlich seiner tatsächlichen Fähigkeiten im Unklaren zu halten. Persönliche Fehleinschätzungen in der Auswahl des Agenten sind daher wahrscheinlich (Problem der „adverse selection“). Für beide Problemfelder gilt es, eine geeignete Vorsorge zu treffen, etwa durch gezielte Governancelösungen, die die Leistungsprozesse des Agenten gut beobachtbar und damit einschätzbar machen bzw. durch maßgeschneiderte Incentivlösungen, die sich stärker am Ergebnis der Aufgabenerfüllung orientieren und so die Interessenslagen zwischen Principal und Agent aneinander angleichen (dazu Fama und Jensen 1983; sowie Eisenhardt 1989). Die Forschungen angeleitet durch die Denkfiguren der Principal-Agent-Theorie haben das Ziel, die hier nur grob geschilderten „agency problems“ in ihren kausalen Wirkungsmechanismen immer genauer zu erfassen und Lösungen zu finden, die die damit verbundenen „agency cost“ möglichst geringhalten, um so die von den Eigentümern gewünschte Effizienz in der Performance des Unternehmens zu gewährleisten (nach wie vor grundlegend dazu Jensen und Meckling 1976).

Da die Principal-Agent-Theorie vornehmlich in der Theoriearchitektur des wirtschaftswissenschaftlichen Denkens begründet ist, ist ihre bis heute prägende paradigmatische Dominanz in den management sciences gut nachvollziehbar. Auf den ersten Blick scheint ihre besondere Erklärungskraft im Kontext von familiengeführten Unternehmen allerdings nicht zum Tragen zu kommen. Überall dort, wo die Einheit von Eigentum und Führung an der Spitze eines Unternehmens noch voll in Wirksamkeit ist, dort dürften eigentlich keine der bekannten „agency problems“ zu beobachten sein (vgl. Jensen und Meckling 1976; Chrisman et al. 2004; Shukla et al. 2014, S. 103).

Ein genaueres Hinschauen der Forschung seit der Jahrtausendwende hat allerdings auch bei Familienunternehmen eine Reihe von Konstellationen zu Tage gefördert, die gerade mit der Brille dieser Theorie in ihrer Problemhaftigkeit gut erfasst werden können. Dazu zählt etwa die Situation, wo die Familie ihren außergewöhnlichen Einfluss an der Unternehmensspitze dafür nutzt, Erträge in einem unangemessenen Ausmaß für die Familie abzuzweigen, Familienmitglieder in der Firma zu bevorzugen, Nachfolger in der Führung zu etablieren, obwohl ihnen die erforderliche Eignung fehlt und ähnliche Phänomene mehr, die man gerne als Nepotismus bzw. als „asymmetric altruism“ bezeichnet (vgl. Madison et al. 2016, S. 68). Ihnen ist gemeinsam, dass die Eigentümer den Familieninteressen einen deutlichen Vorrang gegenüber den Überlebenserfordernissen des Unternehmens einräumen und dieses damit in seiner Entwicklungsfähigkeit nachhaltig schädigen (dazu vgl. etwa auch Shukla et al. 2014, S. 106; sowie Chua et al. 2009).

Zu den typischen Problemfeldern, die speziell aus der Einheit von Eigentum und Führung resultieren können, zählt auch das Phänomen, dass gerade sehr erfolgreiche Unternehmer an der Unternehmensspitze im Zeitverlauf ihr ursprüngliches unternehmerisches Gespür einbüßen und anfangen an Produkten, Dienstleistungen, an Verfahren und Technologien festzuhalten, obwohl in strategischer Hinsicht längst entsprechende Innovationen erforderlich wären. Aus diesen strategischen Versäumnissen erwachsen zumeist auf Sicht gesehen operative Fehlentwicklungen, die in der Regel in existenzgefährdenden Unternehmenskrisen münden. „The emotional attachment the owning family has on the firm may inhibit business decisions decreasing firm performance“ (Madison et al. 2017, S. 60).

Wenn in späteren Phasen des Lebenszyklus von familiengeführten Unternehmen die Einheit von Führung und Eigentum aufgelöst wird, dann entstehen unweigerlich Konfliktdynamiken innerhalb des Gesellschafterkreises, die dazu führen, dass ein einheitlicher unternehmerischer Gestaltungswille auf der Eigentümerseite verloren geht. Dies ist häufig der Fall, wenn sich die Unternehmerfamilie in Stämme aufspaltet und diese Stammesinteressen die Oberhand gewinnen.

Vergleichbare Konfliktdynamiken können zwischen tätigen und nichttätigen Gesellschaftern erwachsen, zwischen Geschwistern in der Führung des Unternehmens, zwischen Mehrheits- und Minderheitsgesellschaftern innerhalb der Unternehmerfamilie und in vergleichbaren Familienkonstellationen. Familienbasierte Konflikte haben in der Regel die Tendenz, sich mangels klarer Autoritätsverhältnisse zu verselbständigen und sich chronisch immer wieder aufzuladen und so die unternehmensbezogenen Entscheidungserfordernisse für sich zu okkupieren (vgl. dazu von Schlippe 2014).

Aus Sicht der Principal-Agent-Theorie eignen sich solche Problemlagen besonders gut, eigentümerbezogene Zielkonflikte (d. h. ein „misalignment of shareholder goals“) auf Dauer zu stellen und damit die Führbarkeit des Unternehmens spürbar zu beeinträchtigen. Sobald die Fähigkeit zu einer einheitlichen Entscheidungsfindung im Eigentümerkreis zerbricht, braucht es Governancelösungen, die das Unternehmen vor den destruktiven Dynamiken auf Eigentümerebene schützen (dazu auch Zellweger und Kammerlander 2015). Die hier geschilderten Beeinträchtigungen erzeugen immer dann in einem besonderen Ausmaß negative Konsequenzen, wenn das Unternehmen komplett von einem Fremdmanagement geführt wird und dieses auf der Seite der Eigentümer kein entscheidungsfähiges Gegenüber besitzt (vgl. Villalonga und Amit 2006).

Angesichts der großen Zahl an Forschungsarbeiten, die mit der Brille dieses Theoriegebäudes inzwischen auf Familienunternehmen schauen, überrascht es nicht, das sich dieser Forschungsstrang in den Annahmen bezüglich seiner Fruchtbarkeit auch für diesen Unternehmenstyp voll bestätigt fühlt. „In conclusion, agency theory is an applicable theoretical perspective for family firm governance and performance research“ (Madison et al. 2017, S. 65). Diese Bestätigung erwächst zudem aus der inzwischen gewonnenen Gewissheit, dass die von der Theorie entwickelten Lösungen für die Bearbeitung der diagnostizierten agency problems auch für familiengeführte Unternehmen eins zu eins passen. In diesem Punkt machen sie zu Nichtfamilienunternehmen keinen Unterschied. Die diesbezüglichen Forschungen zeigen, „that agency governance mechanism, such as the presence of a board of directors, incentive compensation plans, and monitoring activities serve their theorized purpose within family firms“ (Madison et al. 2017, S. 61). Speziell in diesen Dimensionen gibt es also keinen Unterschied zu Nichtfamilienunternehmen, für die diese Lösungsperspektiven ja ursprünglich entwickelt worden sind.

Zusammenfassend lässt sich feststellen, dass sich die bisherige Familienunternehmensforschung, die mit Hilfe der Principal-Agency-Theorie operiert, auf Themenfelder fokussiert, die alle anzeigen, dass diese Unternehmen über kurz oder lang von ihrem ursprünglichen Erfolgskurs, der sich einer spezifischen unternehmerischen Orientierung verdankte, abweichen. Die diesbezügliche Forschung beschäftigt sich also primär mit der „dunklen“ Seite dieses Unternehmenstyps, mit seinen häufig beobachtbaren Selbstgefährdungspotenzialen begleitet von der Vermutung, dass diese Potenziale auf längere Sicht gesehen die Entwicklung dieser Unternehmen bestimmen. Folgt man dem prognostischen Potenzial dieser Forschungsrichtung, das diese selbst für sich beansprucht, so führen diese Selbstgefährdungspotenziale entweder zu einem Verschwinden vom Markt oder durch eine erfolgreiche Übernahme der von der Theorie erarbeiteten Governancelösungen zu einer schrittweisen Angleichung an die Erfolgsmuster von Nichtfamilienunternehmen.

Im blinden Fleck dieser Forschung bleibt allerdings der Umstand, dass eine Vielzahl von familiengeführten Unternehmen über Generationen hinweg ausgesprochen erfolgreich performen und dabei ihren Charakter als Familienunternehmen durchaus bewahren. Es gibt inzwischen ausreichend empirische Forschungen, die diesen Befund belegen (als Beispiel sei auf Anderson und Reeb (2003) sowie Amit und Villalonga (2014) verwiesen). Es gibt zudem eine umfangreiche Forschung zu den besonderen Wettbewerbsvorteilen familiengeführter Unternehmen (für einen Überblick vgl. Xi et al. 2015), die zeigen kann, welche „organizational capabilities“ solche Unternehmen entwickeln, um in ihren Märkten die Nase vorn zu haben (am Bespiel der „hidden champions“ belegt dies Simon 2012b). All diese Faktoren, vor allem aber die spezifischen Führungspraktiken erfolgreicher Familienunternehmen bekommt man mit dem Theorierepertoire des Principal-Agent-Ansatzes nicht zu Gesicht, dies auch deshalb, weil in dieser Theorieperspektive Führung lediglich die schlichte Realisation der unterstellten Ursache/Wirkungszusammenhänge zu ihrem Gegenstand hat. Sie ist keine eigene Gestaltungsgröße in der Unternehmensentwicklung.

### Die Stewardship Theorie

Die Zuspitzungen in den Grundannahmen menschlichen Verhaltens, wie sie in der Principal-Agent-Theorie für das Zusammenspiel der Akteure in Unternehmen vorgenommen worden sind, sind im wissenschaftlichen Diskurs nicht unwidersprochen geblieben. In den zurückliegenden 25 Jahren hat sich eine eigene Gegenrichtung herauskristallisiert, die sich unter dem Titel „Stewardship Theory of Management“ zusammenfassen lässt (als Basisarbeit für diesen Ansatz gilt Davis et al. 1997). Dieser Zugang hat vor allem in der Familienunternehmensforschung erhebliche Resonanz gefunden.

Die Vertreter dieser Denkrichtung greifen im Aufbau eines tragfähigen Begründungszusammenhanges für die Frage, was einem erfolgreichen wirtschaftlichen Zusammenwirken in Unternehmen letztlich zugrunde liegt, auf eine spezifische Kombination psychologischer und organisationssoziologischer Theorieelemente zurück. Auf der Seite der handelnden Akteure unterstellen sie nicht zwangsläufig eine persönliche Nutzenoptimierung. Die individuelle Motivationslage und Verhaltensdisposition des Einzelnen ist im Kontext eines Unternehmens aus Sicht dieses Theoriezugangs durchaus kontingent. Diesbezügliche Festlegungen können in sehr unterschiedliche Richtungen erfolgen. Zurückgreifend auf die McGregor’schen Überlegungen zu den persönlichen Motivkräften in Unternehmen (vgl. McGregor 1960) sehen die Proponenten des Stewardship Ansatzes auch die Möglichkeit, dass Akteure ihre ganz persönliche Erfüllung darin sehen, ihre ganze Kraft und Energie für den Erfolg des jeweiligen sozialen Ganzen einzusetzen (vgl. Donaldson 1990). Diese Art des Antriebs fußt auf der intrinsisch verankerten Motivation, im Sinne der übernommenen Aufgabe erfolgreich wirksam werden zu können und dafür die erforderlichen Kooperationsbedingungen selbst aktiv bereitzustellen, ohne dabei den eigenen, höchstpersönlichen Nutzen ständig vor Augen zu haben. Im Unterschied zur agency theory werden hier Manager als Stewards beschrieben, „whose behavior is based on an intrinsic desire to serve the firm and will therefore naturally align with the principal’s interests“ (Madison et al. 2016, S. 66/67).

Klar ist, dass solche Verhaltensdispositionen nur in einem organisationsinternen Umfeld zum Tragen kommen, das ein diesbezügliches Verhalten stimuliert und „belohnt“. Das braucht zwischenmenschliche Verhältnisse, in denen man sich wechselseitig vertrauen kann, die auf fairen, reziproken Austauschbeziehungen beruhen, bei denen man sich darauf verlassen kann, dass sich alle Beteiligte in ihrem Engagement am gemeinsamen Erfolg des größeren Ganzen orientieren. Vertrauen ist in diesem Kontext zweifelsohne die entscheidende Ressource für ein erfolgreiches Miteinander. Die laufende Erneuerung dieser Ressource braucht eine feste Verankerung in geeigneten organisatorischen Rahmenbedingungen, gemeint ist damit eine stimmige Kohärenz zwischen der täglich erlebten Führungspraxis, den zugrundeliegenden Entscheidungsstrukturen und einer starken Unternehmenskultur, in die das ganze Geschehen eingebettet ist. Ein solcher Rahmen lässt Arbeitsbedingungen erwarten, die für eigenes Entscheiden einen gewissen Freiraum bereitstellen, die eine eigenständige Verantwortungsübernahme gepaart mit einer gewissen Risikobereitschaft belohnen, die auf übermäßige Kontrollmechanismen verzichten und die intrinsische Motivation und die hohe Identifikation mit dem Vorankommen des Unternehmens nicht durch die Betonung ausschließlich monetärer Incentives verdrängen (dazu vor allem Davis et al. 1997). Folgt man diesen Prämissen der Stewardship-Theorie, dann stellt sich Führung primär in den Dienst der Herstellung und Aufrechterhaltung der eben beschriebenen Organisationsverhältnisse, die das erfolgreiche Überleben des größeren sozialen Ganzen ins Zentrum der gemeinschaftlichen Anstrengungen rücken. Gelingt dies, dann setzt das bei den Beschäftigten jenen Energieeinsatz frei, den diese Theorie mit ihrem Menschenbild unterstellt.

Diese Theorieüberlegungen sind bei einem erheblichen Teil der Familienunternehmensforscher und Forscherinnen auf große Resonanz gestoßen (diesbezügliche Literaturreviews bieten etwa Madison et al. (2016) sowie Odom et al. 2019). Manche halten diese geradezu für „ideal for explaining governance in the family business context“ (Davis et al. 2010). Dieselben Autoren betonen, „that family firms foster trust and commitment among employees, making stewardship the ‚secret sauce‘ for creating a competetive advantage in family firms“ (zit bei Madison et al. 2016, S. 69). Studien zeigen, dass überall dort, wo sich Eignerfamilien selbst in den Dienst einer langfristigen Überlebenssicherung des Unternehmens stellen, durchgängig die Ausprägung einer Stewardship-Kultur zu beobachten ist (Le Breton-Miller und Miller 2009). Mit Blick auf die transgenerationale Entwicklungsperspektive von familiengeführten Unternehmen kommen Arrègle et al. (2007) zu einem ganz ähnlichen Ergebnis. Gestützt durch eine solche von der Unternehmensspitze vorgelebte Kultur festigen sich Führungspraktiken, die auf gelingende Kooperation wertlegen, eigenverantwortliches Handeln und engagierte Verantwortungsübernahme fördern und für ein Lebendigbleiben einer hohen Identifikation mit dem Unternehmen sorgen (Eddleston und Kellermanns 2007). „Stewardship orientation is argued to enhance the achievment of long-term organizational goals that emerge from reciprocal altruism, prosocial behavior, and mutual interdependence“ (Eddleston et al. 2008; zit. bei Odom et al. 2019, S. 51). In Summe sind das Unternehmen, die schwer imitierbare Ressourcen und Fähigkeiten hervorbringen, die kontinuierlich wachsen, sich resilient auch in Krisenzeiten bewähren und sich letztlich wirtschaftlich überdurchschnittlich erfolgreich in ihren Märkten behaupten (vgl. Miller und Le Breton-Miller 2005; Miller et al. 2008; sowie Sirmon und Hitt 2003).

Stellt man die beiden geschilderten Theorierichtungen einander gegenüber, so ist bei aller Gegensätzlichkeit ihrer Grundannahmen doch faszinierend, dass beide sichtlich mit einigem Recht feststellen, dass der jeweilige Ansatz gestützt durch eine Vielzahl empirischer Studien gerade für die Besonderheiten von Familienunternehmen absolut passend ist. Die Verfechter der Principal-Agent-Theorie können sich in ihrer Annahme bestätigt fühlen, dass diese Unternehmen nur dann eine gute Zukunftsperspektive haben, wenn sie die empfohlenen Governancelösungen erfolgreich implementieren und sich damit den Erfolgsmustern von Nichtfamilienunternehmen schrittweise annähern. Aber auch die Stewardship orientierte Forschung kann zeigen, dass gerade die langlebigen Familienunternehmen ihre erstaunliche Überlebensfähigkeit dem Lebendighalten von Stewardship-Prinzipien verdanken. Offensichtlich ist das Feld der Familienunternehmen so heterogen, dass es in einer ausreichenden Vielfalt unterschiedliche Beispiele zur Verfügung stellt, so dass die Forschung zu so gegensätzlichen, verallgemeinerungsfähigen Aussagen kommen kann, die die bestehenden paradigmatischen Ausrichtungen jeweils bestätigen und ihre weitere Anwendung ermutigen. Das ist forschungssoziologisch an sich ein spannendes Phänomen. Inzwischen mehren sich die Arbeiten, die diese Gegensätzlichkeit selbst zum Gegenstand von Überlegungen machen und die sich sowohl das Forschungsfeld selbst dahingehend ansehen, warum es dieses Nebeneinander der Gegensätze ermöglicht, als auch der Frage nachgeht, was es an zusätzlicher Theoriebildung braucht, um diese empirischen Unterschiede besser zu erklären (vgl. etwa Madison et al. 2016, 2017; Le Breton-Miller und Miller 2009; sowie Odom et al. 2019). Ein Phänomen zeigt die bisherige Theorierekonstruktion allerdings bereits recht deutlich. Der Zusammenhang von Führung und Organisation diese verstanden als kollektiver Akteur eigenen Typs bleibt in der bisherigen Forschung zu Familienunternehmen auffällig unterbelichtet (vgl. auch die Einschätzung von Xi et al. 2013, S. 123). Vermutlich hängt dies mit dem methodologischen Individualismus, und der Suche nach zeitstabilen Ursache-Wirkungs-Relationen zusammen, die das Denken in den management sciences nach wie vor dominieren. Diesen hier deutlich gewordenen Einseitigkeiten bleibt allerdings auch die nächste zur Zeit sehr prominente Theorieentwicklung, die nun kurz vorgestellt wird, verhaftet.

### Der Social-Emotional-Wealth-Ansatz (SEW)

Der SEW-Ansatz stellt im Grunde genommen eine weiterentwickelte Variante der Principal-Agent-Theorie dar, eine Variante, die im zurückliegenden Jahrzehnt in der einschlägigen Forschung allerdings sehr an Bedeutung gewonnen hat. „SEW has emerged as a major approach for studying the behaviors of family firms“ (Odom et al. 2019, S. 59; zu einer ganz ähnlichen Einschätzung kommen Schulze und Kellermanns 2015; sowie Sharma et al. 2014, S. 5). In seinen theoretischen Grundannahmen greift dieser Forschungsansatz auf Denkfiguren zurück, die im „Behavioral Agency Model“ (BAM) entwickelt worden sind (dazu Wiseman und Gomez-Majia 1998). Im Mittelpunkt stehen Annahmen zu unternehmerischen Erwartungshaltungen im Umgang mit vermuteten Risiken und den daraus abgeleiteten Verhaltenspräferenzen wirtschaftlicher Akteure (fußend auf den vielfach zitierten Arbeiten von Kahnemann und Tversky 1979). Damit kommen unterschiedliche Risikopräferenzen des Principals wie des Agents, die sich im Zeitverlauf durchaus ändern können, sehr viel stärker in den Blick. BAM „suggest selfinterested individuals are more likely to prefer the minimization of current losses over maximizing future gains“ (Shukla et al. 2014, S. 106). Mit Blick auf Familienunternehmen wird weiters von der weithin geteilten Beobachtung ausgegangen, dass die Inhaber mit ihren Unternehmen nicht nur wirtschaftliche Ziele (z. B. einen möglichst hohen Gewinn) verfolgen sondern, dass ihrem unternehmerischen Engagement und den damit verbundenen strategischen Weichenstellungen ein charakteristisches Set an nichtökonomischen Zielsetzungen zugrunde liegt (vgl. Berrone et al. 2012). Dazu zählen das Bedürfnis, als Familie die Kontrolle über die Unternehmensentwicklung zu halten, die gewonnene Reputation und den sozialen Status im Umfeld zu sichern, das Unternehmen für nachfolgende Generationen zukunftsfähig zu halten, die wirtschaftliche Absicherung der Familie zu gewährleisten, etc. Die Kernthese des SEW Ansatzes läuft darauf hinaus, dass die Unternehmerfamilie alles daran setzt und ihre Eigentümerrolle dafür nutzt, um eine Beeinträchtigung ihrer nichtökonomischen Ziele zu verhindern, auch wenn dies auf Kosten der wirtschaftlichen Ertragskraft des Unternehmens geht (erstmals empirisch belegt von Gomez-Majia et al. 2007; ähnlich auch Odom et al. 2019, S. 53). In diesem Sinne wird SEW definiert als „non-economic statisfaction derived from firm ownership“ (Shukla et al. 2014, S. 108).

Inzwischen haben die Hauptvertreter dieses Forschungszugangs elaborierte Messkriterien vorgelegt, an Hand derer empirische Studien die Stichhaltigkeit der Grundannahmen des Ansatzes erhärten können (dazu Berrone et al. 2012); zusammengefasst in den 5 FIBER Dimensionen (**F**amily controll and involvment; **I**dentifikation of family members with the firm; **B**inding social ties; **E**motional attachment of family members to their firm; **R**enewal of family bonds to the firm through dynastic succession; dazu auch Odom et al. 2019, S. 53/54). In jüngster Zeit sind dadurch tatsächlich eine Fülle von Studien angestoßen worden, die zu belegen versuchen, dass in allen Fällen, in denen die Eignerfamilie ihren unternehmerischen Einfluss hat aufrechterhalten können, dieser Einfluss dazu genutzt wird, auf Basis eines typischen Risikovermeidungsverhaltens den nichtökonomischen Zielen zum Durchbruch zu verhelfen, ein Umstand der in der Regel auf lange Sicht die Ertragskraft des Unternehmens schädigt (einen Überblick über diese Forschungsanstrengungen geben Neacsu et al. 2017). Diese nachteiligen Konsequenzen betreffen die Innovationsfähigkeit, die Möglichkeiten zu einer risikoadäquaten Diversifikation der unternehmerischen Aktivitäten, die Rekrutierung geeigneter Führungskräfte, die wettbewerbsfähige Allokation von Ressourcen und ähnliches mehr. Um solchen Entwicklungen vorzubeugen, wird die konsequente Implementierung jener Governancemechanismen erforderlich, wie sie in der Forschungstradition der Principal-Agent-Theorie erarbeitet worden sind.

Es zählt zu den bislang noch wenig hinterfragten Glaubenssätzen des SEW Ansatzes, dass die Verfolgung nichtökonomischer Zielsetzungen, die dem primären Interesse der Unternehmerfamilie entspringen, in einem unmittelbaren Gegensatz zur Fokussierung auf die Ertragskraft des Unternehmens steht. Diese Grundüberzeugung entspricht zur Gänze den Annahmen, wie wir sie auch bei der Principal-Agent-Theorie antreffen. Je ungebremster sich der Einfluss der Unternehmerfamilie auf die Entwicklung des Unternehmens entfalten kann, umso wahrscheinlicher ist es, dass dieser Einfluss zu Lasten der Leistungsfähigkeit des Unternehmens geht. Inzwischen gibt es jedoch eine ausreichende empirische Evidenz, dass dem nicht so ist. Es ist gerade die Einbettung der wirtschaftlichen Dimension der Unternehmensentwicklung in eine nichtökonomische, dem familialen Background entnommene Wertebasis, die diese Unternehmen auf längere Sicht auch wirtschaftlich besonders erfolgreich macht (vgl. Wimmer (2021) aber auch die ganze Diskussion um die einzigartigen Ressourcen dieser Unternehmen; beispielhaft Sirmon und Hitt 2003; und Rau 2014). Dieses synergieerzeugende Zusammenspiel zwischen nicht ökonomischen und unmittelbar wirtschaftlichen Zielsetzungen, ein Umstand, der direkt das Resultat einer breiter angelegten Sinnfundierung unternehmerischen Handelns ist, kommt mit dem Denkrepertoire des SEW-Ansatzes nicht in den Blick (so auch Shukla et al. 2014, S. 114).

Deshalb ist mit Blick auf den SEW-Ansatz der Forderung „a more fine-grained theoretical and empirical research is still necessary“ (Neacsu et al. 2017, S. 154) nur beizupflichten.

### Zentrale Schlussfolgerungen aus diesen Theoriereviews

Die kurze Rekonstruktion verschiedener Theorieperspektiven auf Familienunternehmen hat gezeigt, dass diese auf einem Set von Annahmen beruhen, die rund um die Interessen und Motivlagen der handelnden Akteure kreisen. Akteure sind hier individuelle Rolleninhaber (Eigentümer, Manager) bzw. Familien in ihrer Funktion als Eigner am Unternehmen, dieses verstanden als ein spezifisches Geflecht an Vertragsbeziehungen, das die daran Beteiligten für ihre jeweilen Zwecke und Interessen nutzen wollen. Das Unternehmen als soziale Einheit, als korporativem Akteur ganz eigenen Typs kommt bei diesen Theoriekonstruktionen (auszunehmen ist hier der Stewardship Ansatz) lediglich in einem instrumentellen Sinne vor, als ökonomische Realität, die im Ergebnis dem einen oder anderen der beteiligten Akteure einen Nutzen stiftet. Das Unternehmen als kollektiver Akteur, als eigenständige Überlebenseinheit mit einer spezifischen hochkomplexen Eigendynamik ist in diesen Betrachtungsweisen kein eigenes Thema. Diese Realitätsdimension bleibt gleichsam im blinden Fleck der Beobachtung, ein Umstand, der sich primär dem methodologischen Individualismus dieser wirtschaftswissenschaftlichen Denktraditionen verdankt. Die Fokussierung auf die persönliche Interessenslage individueller Akteure und ihrer Beziehungen untereinander lässt kein angemessenes Verständnis für die spezifische Systemhaftigkeit des Unternehmens wie auch der Familie entstehen. Ähnliches gilt für das Phänomen Führung. Auch dieses wird primär als ein Ausfluss persönlicher Motivlagen verstanden. Führung wird in diesem Sinne als ein Set an persönlichen Eingriffsmöglichkeiten konzipiert, die den Akteuren zur Verfügung stehen, um das Geschehen im Unternehmen in Richtung des eigenen Nutzens zu lenken.

Es ist die These des vorliegenden Beitrags, dass mit diesen erkenntnistheoretischen Begrenzungen der wirtschaftswissenschaftlichen Forschung wesentliche Dimensionen der Eigenart von familiengeführten Unternehmen nicht in den Blick kommen. Dieses Erkenntnisdefizit wird inzwischen von Teilen der Familienunternehmensforschung ganz ähnlich beschrieben (vgl. etwa Sharma et al. 2014). Um diese spezifische Systemqualität organisierter wirtschaftlicher Akteure in unserer Gesellschaft adäquat beschreibbar zu machen, dazu verhelfen eine Reihe aus der Systemtheorie gewonnener Denkfiguren, die in den zurückliegenden drei Jahrzehnten das Verständnis von Führung und Organisation angereichert haben (ausführlicher dazu Wimmer 2012). Diesem Aspekt sind die abschließenden Überlegungen gewidmet.

## Eine systemtheoretische Perspektive auf die Eigenart von Führung und Organisation von Familienunternehmen

Es gibt eine wenn auch noch junge Forschungstradition, die von der Überzeugung getragen wird, dass sich das Theorierepertoire der neueren Systemtheorie gerade für das Erfassen der komplexen Besonderheiten von Familienunternehmen und ihrer spezifischen Funktion in unserem Wirtschaftssystem besonders gut eignet (im Detail Frank et al. 2010; von Schlippe 2013; und Simon 2012a). Wie bereits betont handelt es sich hier um einen in vielerlei Hinsicht eigenständigen Typus von Wirtschaftsorganisationen, der sich in einer großen Vielfalt an Erscheinungsformen auf dem Weg in die Moderne in den zurückliegenden 250 Jahren als eigene Spielart kapitalistischen Wirtschaftens gefestigt und bis heute als überaus lebensfähig behauptet hat (aus historischer Sicht vgl. etwa Colli (2003) und Berghoff (2016) sowie James (2006)).

Aus dieser Theorieperspektive sind Organisationen wie auch Familien in der uns heute vertrauten Form ein Produkt der Moderne, d. h. ein Ergebnis des gesellschaftlichen Strukturwandels, der sich speziell in Europa seit dem 18. Jhd. vollzogen hat (Luhmann 2000, 1990a, 1997, 2020). Unsere heutige Gesellschaft fußt in ihrer Funktionsweise auf einem extrem eigensinnig operierenden und damit auch störungsanfälligen Netz an unterschiedlichsten Organisationen, die ihren Existenzgrund daraus gewinnen, für ihre relevanten Umwelten stets in einem komplexen, netzwerkbasierten Zusammenspiel mit anderen geeignete Problemlösungen bereitzustellen, in diesem Sinne also „wertschöpfend“ tätig zu werden. Die Existenszberechtigung von Organisationen jedweden Zuschnitts liegt also in ihrer Fähigkeit, für ihre Abnehmer, Kunden, Zielgruppen Leistungen anzubieten, die für diese tatsächlich eine mehr oder weniger „wertschaffende“ Lösung darstellen. Die dafür erforderliche Organisationsförmigkeit zeigt sich unter anderem in der Ausprägung spezifischer Mitgliedschaftsrollen, in denen die primär aufgabenbezogenen Verhaltenserwartungen konditioniert werden. Dazu zählt auch die Erwartung an Mitgliedschaft, die hierarchiegeprägten Steuerungs- und Koordinationsmechanismen zu akzeptieren. Organisationen sind in diesem Verständnis ausgesprochen eigensinnige, sich selbst organisierende, hochkomplexe soziale Entitäten, die ihre, für sie konstitutiven Umwelten ständig nach Gelegenheiten abtasten, um durch das Aufgreifen von „Ungelöstem“, durch die gelungene Bearbeitung von an sich unvereinbaren Zielkonflikten das eigene Weiterexistieren wahrscheinlicher zu machen. Dieser eigensinnige Modus der Selbstreproduktion, die in einer kontinuierlichen Verkettung von Entscheidungsprozessen zur Bewältigung von Unsicherheit erfolgt, verbietet es, Organisationen als bloße Werkzeuge in der Hand organisationsextern gedachter Entscheidungsträger (wie bei der Principal-Agent-Theorie) zu konzipieren.

„Was tut ein Manger in einem sich solchermaßen selbstorganisierenden System?“ (Luhmann 1990b). Luhmann konnte auf diese von ihm selbst gestellte Frage keine zufriedenstellende Antwort geben. Die Proponenten der systemtheoretisch fundierten Organisationstheorie haben sich mit dem Phänomen Führung und Management durchgängig schwer getan (ausgenommen Baecker 2008, 2011; sowie Wimmer 2009, 2012). Einen ganz eigenständigen Theoriestrang repräsentiert in diesem Zusammenhang das St. Galler Managementmodell (den aktuellen Stand dazu bieten Rüegg-Stürm und Grand 2020). Das systemtheoretisch fundierte Verständnis von Führung setzt an dem schon geschilderten Verhältnis von Organisationen zu ihren relevanten Umwelten an. Organisationen sind in den Fortsetzungsbemühungen ihrer historisch gewachsenen Identität darauf angewiesen, dass ihre Leistungen tatsächlich eine Lösung für die in ihrem Außen aufgegriffenen Problemlagen darstellen. Diese „Passung“ ist nicht zuletzt deshalb ständig gefährdet, weil Organisationen dazu neigen, sich primär mit sich selbst zu beschäftigen und ihre Umweltwahrnehmungen genau auf diese Tendenz auszurichten. Exakt an diesem Dilemma setzt die Funktion von Führung an.

In einem systemtheoretischen Verständnis von Organisationen kann man davon ausgehen, dass diese in ihrem Inneren eine eigene Funktion ausdifferenzieren, die darauf spezialisiert ist, die Leistungsfähigkeit als Gesamtsystem speziell mit Blick auf ihre relevanten Umwelten kontinuierlich im Auge zu haben.

Organisationen prägen in ihrem Inneren ein eigenes Aufmerksamkeitspotential und die damit verbundenen Verantwortlichkeitszuordnungen aus, die diese selbst im Hinblick auf die Frage im Blick haben, wie es um die eigene Funktionstüchtigkeit als Organisation bestellt ist, vor allem ob und wie die eigene Antwortfähigkeit auf die speziellen Herausforderungen in den relevanten Umwelten gesichert und (wenn erforderlich) weiterentwickelt werden kann. Diese spezifische Verantwortungsdimension nennen wir Führung.

Die Wahrnehmung dieser Funktion sowie die Bearbeitung der damit verbundenen Entscheidungserfordernisse sorgt dafür, dass das System kontinuierlich mit den notwendigen lebenswichtigen Entwicklungsimpulsen versorgt wird. Führung in diesem Sinne passiert also primär von Aussen nach Innen (vgl. Baecker 2008). Sie sorgt dafür, dass es organisationsintern zu realitätsangemessenen Einschätzungen der externen Gegebenheiten speziell der charakteristischen Problemlagen der Abnehmer der eigenen Leistungen kommt und dass daraus geeignete Orientierungen und Impulse für die Gestaltung der internen Strukturen und Prozesse gewonnen werden. Mit anderen Worten, Führung hat es zu ihrem Kerngeschäft, die existenzbegründenden Komplexitäten im Inneren wie im Aussen und die damit unweigerlich verbunden Ungewissheiten bearbeitbar zu machen, d. h. Unsicherheiten erfolgreich in orientierungsstiftende Routinen zu verwandeln und wenn erforderlich diese wieder aufzulösen und durch neue zu ersetzen. Durch diese kontinuierlich erbrachte Leistung der Unsicherheitsbearbeitung erzeugt Führung genau jenen Mehrwert in Organisationen, der die operativ Tätigen entlastet und ihnen den Rücken frei hält, um sich voll im Zusammenspiel mit anderen ihren fachlichen Aufgabenfeldern widmen zu können.

Das hier in aller Kürze vorgestellte Führungs- und Organisationsverständnis versteht Führung als eine spezifische Funktion im Reproduktionsgeschehen von Organisationen. Als solche realisiert sie sich im alltäglichen Kommunikations- und Entscheidungsgeschehen, in denen die diesbezüglichen Aufgabenstellungen prozessiert werden. Diese führungstypischen Aufgabenstellungen speisen sich aus organisationsspezifischen Unsicherheitsquellen wie es beispielsweise alle Fragen nach den strategischen Orientierungen für die Zukunft darstellen oder Fragen zur Bewältigung der Ressourcenknappheit, Fragen nach der Gestaltung der internen Strukturen und Prozesse, Fragen zur Lösung der vielfältigen Personalthemen, Fragen zur ständigen Erneuerung des eigenen Leistungsportfolios und letztlich auch alle Fragen zur nachhaltigen Sicherung der eigenen Ertragskraft. Die mit der Bearbeitung dieser Fragen verbundene Verantwortung ist in Organisationen zumeist an unterschiedlichen Stellen gebündelt. In ihrer jeweiligen Verantwortungsverteilung spiegeln diese Führungspositionen gesamthaft den binnenorganisatorischen Aufbau mit seinen verschiedenen Hierarchieebenen wider. In ihrem spezifischen Zusammenwirken versorgen diese Funktionsinhaber die Organisation und ihre Subeinheiten (mehr oder weniger erfolgreich) mit den erforderlichen Führungsleistungen. An der Art und Weise, wie diese Führungsstrukturen am effizientesten zu gestalten sind, setzen regelmäßig weitreichende Reformbemühungen an. Aktuell sind eine Vielzahl von Veränderungsinitiativen beobachtbar, die darauf abzielen, die Wahrnehmung der Führungsaufgaben auf eine mehr oder weniger „hierarchiefreie“ Weise zu organisieren.

Diese organisationstheoretisch begründete Perspektive unterscheidet sich grundlegend vom nach wie vor dominanten Mainstream der Führungsforschung, der Führung als ein Phänomen sieht, das in erster Linie mit den handelnden Führungspersonen etwas zu tun hat, entweder als Ausdruck ihres besonderen Charismas oder als Bündel aus Begabungen und lebensgeschichtlich erworbenen Kompetenzen (Genaueres dazu Böhmer 2014). Führung realisiert sich hier als gestaltende Einflussnahme von Persönlichkeiten auf ihr organisationales Umfeld, eine Einflussnahme, mit der dieses Umfeld für die Erreichung ihrer Ziele zu formen versucht wird.

Blickt man nun auf das spezifische Führungsgeschehen in familiengeführten Unternehmen, dann bietet sich ein solches an persönlichen Merkmalen festgemachtes Führungsverständnis geradezu an. Wie anfänglich bereits ausgeführt gehört diese Personenorientierung zur unverbrüchlichen DNA dieses Unternehmenstyps. Solche Unternehmen haben in der Regel familiale Grundmuster in ihr Binnengeschehen inkorporiert. Nun sind Familien soziale Systeme, die sich in ihrem internen Kommunikationsgeschehen um das Persönliche ihrer Mitglieder herum reproduzieren. Aus diesen persönlichen Dimensionen schöpfen sie die sie hauptsächlich bewegenden Themenstellungen. Deshalb sind mentale Modelle, die das Führungsgeschehen im Unternehmen in erster Linie an persönlichen Qualitäten festmachen und dabei mit klassischen familialen Autoritätsvorstellungen operieren, in diesem Unternehmenstyp so unmittelbar anschlussfähig. Diese Koinzidenz einer Mainstream geprägten Führungsdenke mit den spezifischen Führungsmustern in Familienunternehmen macht es so verdammt schwer, die Organisation selbst als eigenständiges soziales Phänomen zu sehen und nicht nur Personen im Blick zu haben. Noch viel weniger anschlussfähig ist die Vorstellung, Führung als eine explizite Funktion dieser Organisation zu sehen, die arbeitsteilig von dazu jeweils passenden Funktionsträgern realisiert wird. Die Wahrnehmung von Führungsaufgaben passiert wie hier gesagt implizit, stets eingebettet in das operative Arbeitsgeschehen und verbunden mit der Gewissheit, dass alle existentiell bedeutsamen Entscheidungsmaterien an der Spitze gut versorgt sind.

Will man diese auffällige Personenorientierung familiengeführter Unternehmen organisationstheoretisch beschreibbar machen, dann lohnt es sich auf eine bekannte Luhmannsche Denkfigur zurückzugreifen: gemeint ist sein Begriff der Entscheidungsprämissen (Luhmann 2000, S. 222 ff.).

Luhmann geht davon aus, dass das alltägliche Entscheidungsgeschehen in Organisationen eine vorgelagerte Einrahmung besitzt, die die jeweilige Entscheidungssituation in ihren Optionen eingrenzt und damit für diesen Moment Komplexität reduziert. Bei diesen Prämissen handelt es sich um generelle organisationsintern erzeugte Voraussetzungen, d. h. um allgemeine Orientierungspunkte, „die bei ihrer Verwendung nicht mehr geprüft werden“ (Luhmann 2000, S. 222). Sie legen das konkrete Entscheiden nicht fest, aber sie machen beobachtbar, ob sich dieses innerhalb des Sets an Prämissen bewegt oder nicht. Luhmann unterscheidet drei unterschiedliche Kategorien von Prämissen, die als solche selbst entscheidbar sind: Prämissen, die sich im weiteren Sinne auf den Daseinszweck der Organisation, auf ihre Aufgabenidentität beziehen; Prämissen die sich auf die Kompetenzverteilung im Inneren und auf die diesbezüglichen Koordinations- und Kooperationserfordernisse für eine erfolgreiche Leistungserbringung konzentrieren und letztlich Prämissen, die Entscheidungen den Eigentümlichkeiten der handelnden Personen zugerechnen. Diese drei Entscheidungsprämissen bedingen sich in vielfacher Weise wechselseitig und können je nach den Gegebenheiten einer Entscheidungssituation in unterschiedlichem Ausmaß Priorität gewinnen. Ihnen gemeinsam ist, dass über sie entschieden werden kann. Dann geht es um strategische Festlegungen, um Fragen des Organisationsdesigns oder um bedeutsamere Personalentscheidungen bzw. um das, was in der Organisation mit diesen personenbezogenen Entscheidungen an Erwartungen des damit Festgelegtseins verbunden wird. Ergänzt werden diese drei Kategorien von Entscheidungsprämissen um eine vierte, nämlich um nichtentscheidbare Prämissen, die Luhmann unter dem Begriff der Organisationskultur zusammenfasst (Luhmann 2000, S. 239 ff.). Diese Art von entscheidungswirksamen, kulturbasierten Mechanismen der internen Verhaltenskoordination bilden und verfestigen sich gleichsam hinter dem Rücken der Akteure. In ihnen kondensieren die gemeinsam geteilten Grundüberzeugungen, Werthaltungen und die vielen Selbstverständlichkeiten, die das Miteinander in der Organisation prägen und die letztlich ihren unverwechselbaren, individuellen Charakter ausmachen, ohne dass man sich darüber explizit verständigen müsste (Luhmann ist hier ganz nah am Kulturkonzept von Ed Schein).

Blickt man mit dieser Begrifflichkeit auf familiengeführte Unternehmen, so sticht unmittelbar ins Auge, dass es sich hier um einen Organisationstypus handelt, bei dem die personenbezogenen Prämissen die drei entscheidbaren Kategorien klar dominieren, diese gleichsam in sich einschließen. Man schaut primär auf entscheidungsrelevante Personen und auf das, was diesen als wichtig zugeschrieben wird und gewinnt daraus die relevanten Orientierungspunkte für das alltägliche Entscheidungsgeschehen. Koordination erfolgt über das wechselseitig geteilte Personenwissen, das sich durch langjähriges Zusammenwirken gefestigt hat. In diesem Sinne kann man das Miteinkalkulieren anderer und ihrer Handlungsperspektiven ohne viel Abstimmungsaufwand unterstellen. Strategiethemen, Fragen des Organisationsdesigns ähnlich wie auch die explizite Ausformung des Führungsgeschehens sind implizit in der Personenorientierung mitverpackt, ein Umstand der den Grad der Austauschbarkeit von Personen in familiengeführten Unternehmen deutlich senkt und deren geringe Fluktuationsrate miterklärt. Solange mit diesen impliziten Koordinations- und Steuerungsmechanismen die Leistungsfähigkeit des Unternehmens als Ganzes aufrecht erhalten werden kann, ist die explizite Beschäftigung mit Führung und Organisation kein Thema.

Eine explizite Ausdifferenzierung der Aufgabenfelder von Führung und die damit verbundenen Verantwortungszuordnung auf Stelleninhaber wird erforderlich, sobald die Einheit von Eigentum und Führung aufgelöst wird und wesentlich komplexere Führungs- und Organisationsverhältnisse implementiert werden müssen. Dann wird Führung und die damit verbundenen Verantwortlichkeiten explizit ein Thema. Das was die Funktion von Führung für das Unternehmensganze leistet, tritt dann aus den impliziten familienhaften Versorgungserwartungen an die Unternehmensspitze heraus und muss unternehmensintern dezidiert als ein mit einer eigenen Professionalität zu erbringendes Aufgabenfeld organisiert werden. Dieses Explizitwerden von Führung geht unweigerlich Hand in Hand mit der Implementierung einer stärkeren Organisationsförmigkeit in der Ausgestaltung der internen Strukturen und Prozesse. Das alles zusammen stößt einen weitreichenden und äußerst langwierigen Umbau der betroffenen Familienunternehmen an, ein Umbau, der den angestammten Charakter als familiengeführtes Unternehmen immer wieder von neuem grundlegend auf den Prüfstand stellt.

## Resümee

Zusammenfassend lässt sich die Frage beantworten, worin denn der Geländegewinn besteht, wenn man mit einer systemtheoretischen Brille auf das Führungsgeschehen in Familienunternehmen schaut. Man bekommt unter Führungs- und Organisationsgesichtspunkten den spezifischen Charakter patriarchaler Verhältnisse zu fassen. Man kann die außergewöhnliche, für Außenstehende oft überraschende Leistungsfähigkeit dieser Verhältnisse gut beschreiben aber auch die Entwicklungen genau benennen, unter denen sich diese Verhältnisse destabilisieren und in eine schwer aufzuhaltende selbstdestruktive Dynamik kippen. Deshalb ist dieser Theoriezugang von großem Nutzen, wenn es darum geht, vorausschauend und proaktiv den Transformationsprozess in eine postpatriarchale Führungs- und Organisationswelt zu konzipieren und seine Realisierung erfolgreich zu steuern. Für diese Herausforderungen kann die etablierte Familienunternehmensforschung zur Zeit noch keine adäquaten Antworten bieten (Wimmer 2022).
